# Mechanical Properties and a Constitutive Model of 3D-Printed Copper Powder-Filled PLA Material

**DOI:** 10.3390/polym13203605

**Published:** 2021-10-19

**Authors:** Qing Ji, Zhijun Wang, Jianya Yi, Xuezhi Tang

**Affiliations:** College of Mechatronics Engineering, North University of China, Taiyuan 030051, China; sxwn_jiqing@163.com (Q.J.); yjy2020@nuc.edu.cn (J.Y.); ha_txz@126.com (X.T.)

**Keywords:** 3D printing polylactic acid, copper powder filled polymer, static and dynamic mechanical properties, ZWT constitutive model

## Abstract

Three-dimensional printing is becoming increasingly popular because of its extensive applicability. However, printing materials remain limited. To determine the mechanical properties of polylactic acid (PLA) and copper powder-filled polylactic acid (PLA-Cu) materials subjected to static and dynamic loading, stress–strain curves were obtained under the conditions of different strain rates using a universal material testing machine and a separated Hopkinson pressure bar experimental device. Scanning electron microscopy (SEM) was used to analyze the micro-morphology of the quasi-static compression fracture and dynamic impact sections. The results revealed that the yield stress and elastic modulus of the two materials increased with increasing strain rate. When the strain rate reached a critical point of 0.033 s^−1^, the rate of crack propagation in the PLA samples increased, resulting in the material undergoing a change from ductile to brittle. The strength of the material subjected to dynamic loading was significantly higher than that subjected to quasi-static loading. The SEM image of the PLA-Cu material revealed that copper powder was evenly distributed throughout the 3D-printed sample and that stress initially began to concentrate at the defect site corresponding to the interface between the copper powder and PLA matrix; this resulted in comparatively lower toughness. This finding was consistent with the photographs captured via high-speed photography, which confirmed that the destruction of the specimen was accompanied by an explosive crushing process. Additionally, a Zhu–Wang–Tang constitutive model was used to fit the experimental results and establish a viscoelastic constitutive model of the material. By comparing the dynamic stress–strain curve to the theoretically predicted curve, we found that the established constitutive model could predict the mechanical properties of the PLA-Cu material with reasonable accuracy when the strain was below 7%.

## 1. Introduction

3D printing, also known as additive manufacturing, is a group of techniques that join materials to make objects from 3D model data, therefore, three-dimensional printing technology makes it possible to manufacture complex components that were previously impossible to manufacture via traditional methods [1,2,3,4,5]. The polymer materials used for 3D printing is also becoming increasingly expansive. Acrylonitrile butadiene styrene plastic (ABS) and polylactic acid (PLA) are commonly implemented in 3D printing because of their mechanical properties [6,7,8,9,10,11].

Fused deposition modeling (FDM), is a type of material extrusion additive manufacturing technique whereby the thermoplastic filament is melted by a liquefier and then extruded through a nozzle onto a platform. As the filament is deposited continuously on the print platform, it cools and solidifies while forming bonds with the surrounding materials, producing the 3D objects [12]. FDM printing has demonstrated its ability to print other thermoplastics, including polycarbonate, polystyrene, polyamide, polyether imide, and polyether ether ketone [13]. FDM is used as an effective additive manufacturing method in comparison with SLA, DLP, or SLS, mainly due to its inexpensive and repeatable process, as well as much less post-processing requirements [5].

Unlike the properties of single metals or polymers, the properties of metal powder-filled polymers are comprehensive, and have thus been widely used in the fields of polymer material modification and powder injection molding. Moreover, changing the powder type and/or filling amount can be performed to modify the mechanical properties of the materials. As an example, Antoniac et al. [14] used PLA, Mg, and Vitamin E to prepare anterior cruciate ligament screws that can be used in medical treatment; they verified the feasibility of magnesium-containing PLA biocomposites as raw materials for additive manufacturing by performing experiments. Hanemann et al. [15] modified ABS by introducing BaFe_12_O_19_ as a filler; they found that the addition of the filler reduced the ultimate stress value of the material, relative to that of the pure polymer, while also increasing the magnetic polarization of the material. Mansour et al. [16] prepared graphene-containing PLA samples by following the FDM method and subsequently measured their dynamic mechanical properties. Their experimental results showed that the filling of multifunctional graphene not only yielded more desirable modulus, strength, and hardness values for the 3D-printed nanocomposites, but also increased the damping ratio of the PLA specimens.

In this study, the static and dynamic mechanical properties of PLA and copper powder-filled PLA material (PLA-Cu) were prepared using a 3D printer. The static and dynamic mechanical properties of the two materials were tested by employing a universal material machine and an experimental separated Hopkinson pressure bar (SHPB) device; the stress–strain curves for the two materials were subsequently obtained to explain the failure and deformation behavior of the two materials under different strain conditions. A Zhu–Wang–Tang (ZWT) nonlinear viscoelastic constitutive model that can describe the viscoelastic deformation range of the polymer was established based on the experimental results. Lastly, scanning electron microscopy (SEM) was used to microscopically characterize the 3D-printed samples, as well as the static and dynamic loading-induced fractures.

## 2. Experimental Methods

### 2.1. Experimental Materials

Material: PLA wire rod, diameter 2.85 m, Ultimaker Company, Utrecht, The Netherlands. Specific parameters are shown in Table 1 and Table 2.

### 2.2. Experimental Equipment

The experimental equipment information is shown in Table 3.

### 2.3. Specimen Preparation

**Figure 1:**
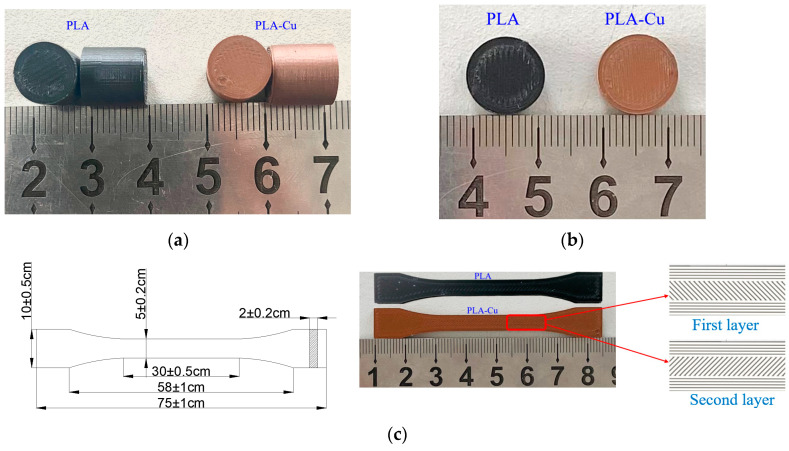
Specimen size and physical drawing. (**a**) Quasi-static compression; (**b**) dynamic compression; (**c**) quasi-static stretching.

To analyze the influence of copper powder on the mechanical properties and microstructure of PLA-based materials, quasi-static tensile, compression, and SHPB experiments were conducted to elucidate the basic mechanical properties of the materials. Three different specimens were prepared using a 3D printer. The size of the tensile specimen is shown in Figure 1c; the size of the static compression specimen was 10 mm × 10 mm. Based on the stability of the sample and the uniformity of the stress wave passing through the low-wave impedance material, the size of the dynamic impact sample was 12 mm × 4 mm. The sample size and physical maps are shown in Figure 1.

All samples were stacked layer by layer in grid cross-filling mode, as shown in Figure 1c.

The experimental samples were printed at room temperature using an Ultimaker S5 3D printer. The model was constructed using CAD software, and the STL file was exported. The STL file was imported into Cura, i.e., the open-source slicer software of the Ultimaker S5 3D printer, to set the slicing and printing parameters. During the printing process, the filling density of the 3D print was set to 100% to minimize the number of internal voids. The PLA printing melting temperature range was approximately 190–230 °C; therefore, the printing temperature of the specimen was set at 210 °C, and the temperature of the printing support platform was set at 60 °C to ensure that the specimen could be firmly attached to the supporting platform during the printing process. Table 4 lists the fixed parameters.

### 2.4. Experimental Theory

Quasi-static tensile and compression experiments were performed on the PLA and PLA-Cu materials using a WDW-20 electronic universal material testing machine. The length of the gauge distance section of the small tensile sample was set as L_0_ = 25 mm, and the tensile and compression velocities were 2, 5, 10, 20, and 50 mm/min. The stress–strain curve for each material was derived from the obtained force-displacement curve [15,17].

**Figure 2:**
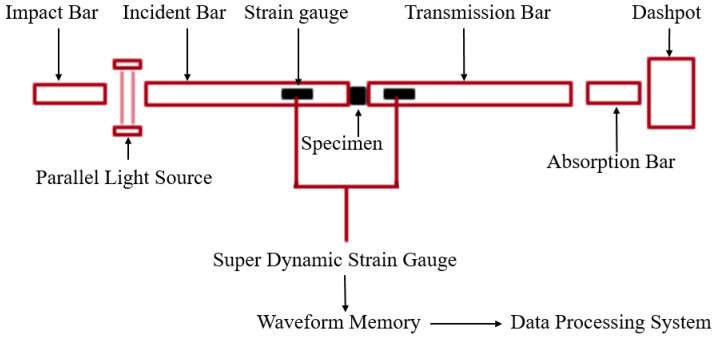
Schematic diagram of SHPB experimental device.

The SHPB device was used to perform impact loading experiments on the PLA and PLA-Cu materials. A schematic of the SHPB experimental device is shown in Figure 2. By applying different air chamber pressures, the initial velocity at which the impact bar collided with the incident bar could be controlled, a 1D stress compression wave could be generated in the incident bar, and the propagation of the stress wave in the bar could be monitored using strain gauges attached to the incident and transmission bars. A dynamic strain gauge and a data acquisition system were used to collect and record the information.

The gains of the incident wave and transmitted wave of the dynamic strain gauge were set to 500 and 1000 times, respectively. The incident, reflected, and transmitted waves were amplified to facilitate acquisition and processing. Lastly, the two-wave method was adopted for data processing [18,19,20].

## 3. Results and Discussion

### 3.1. Quasi-Static Experimental Results

**Figure 3:**
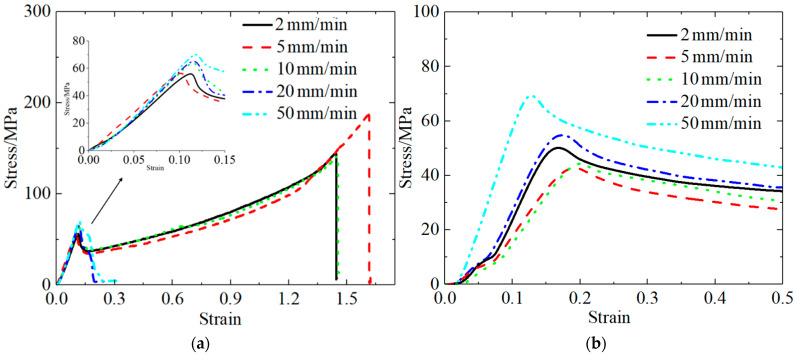
Quasi-static stress–strain curve of PLA material. (**a**) Tensile stress–strain curve; (**b**) compression stress–strain curve.

Table 5 and Table 6 present the PLA quasi-static experimental results at different tensile and compression rates. The stress–strain curves for the PLA materials were obtained under the conditions of different tensile and compression rates by processing the experimental data, as shown in Figure 3.

In the table, b and d represent the length and width of the cross section of the stretched pattern, while r and h represent the cross-sectional radius and compression height of the compression pattern. The loading speed and strain rateS used in the tensile test are shown in Table 5, Table 6, Table 7 and Table 8, which WERE consistent with Yi Jianya [21] and Liu Tongxin [22].

Because there were some inevitable small differences in manual operations, and defects in the sample manufacturing process in the experiment, in order to ensure the accuracy of the experimental data, each experiment was repeated three times, and one of the three test datasets was selected to draw the stress–strain curve. The errors of the experimental data are given in Table 5, Table 6, Table 7 and Table 8.

Figure 3 shows the quasi-static stress–strain curves for the PLA material. As can be seen in Figure 3a, the shape of the tensile curve for the PLA material significantly depended on the rate at which tension was applied, implying that the material exhibited the strain rate effect. As the strain rate increased, the strength of the material increased, the toughness and ductility decreased, and the material exhibited characteristics indicative of a change from tough to brittle.

When the rate of applied tension was less than or equal to 10 mm/min, the PLA tensile curve resembled a typical tensile stress–strain curve for a glassy polymer. In the initial loading stage, the stress and strain increased. Before the yield point was reached, the stress and strain become proportional, conforming to Hooke’s law. The initial elastic modulus of the PLA material was obtained by fitting the stress–strain curve in the elastic stage, i.e., E = Δε/Δσ. The deformation at this stage was the general elasticity associated with the growth of the internal bonds of the polymer and the change in the bond angle.

The material continued to stretch beyond the yield point, and the strain continued to increase within a certain range under the condition that the stress decreased. The deformation at this stage was forced high elastic deformation, which was induced by the imposition of the force and chain movements. When the strain exceeded 15%, the stress increased again; this phenomenon was attributed to the macromolecule orientation along the direction of the force field created by the movement of the chain segment. Finally, the PLA material reached the breaking point, and its elongation at break reached 162% when the rate of applied tension was 5 mm/min, and the maximum tensile strength was 186.99.

As demonstrated by the tensile stress–strain curve in Figure 3a, the PLA material did experience a strain-hardening stage when the rate of applied tension exceeded 10 mm/min; however, beyond the yield point, the stress decreased as the strain increased. Additionally, only a small amount of deformation occurred prior to material fracture; beyond this point, the shape of the stress–strain curve was found to be irregular until the specimen fractured. This indicates that the fracture toughness of the PLA material significantly decreased with the increasing rate of applied tension.

Figure 3b shows the quasi-static compression stress–strain curve for the PLA material. The characteristics of the quasi-static compression curve were consistent with those of a hard and tough polymer material, which are characterized by a large modulus and high yield point.

**Figure 4:**
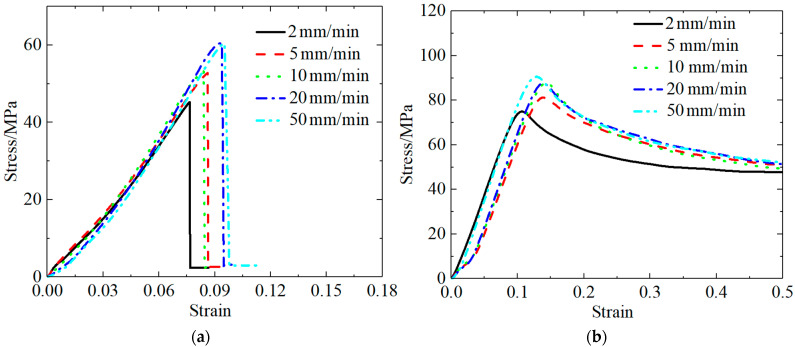
Quasi-static stress–strain curve of PLA-Cu material. (**a**) Tensile stress–strain curve; (**b**) compression stress–strain curve.

Table 7 and Table 8 present the quasi-static experimental results for the PLA-Cu material at different tensile and compression rates. The stress–strain curves for the PLA-Cu material were obtained under the conditions of different tension and compression rates after processing the experimental data (Figure 4).

Figure 4a shows the quasi-static tensile stress–strain curves for the PLA-Cu material subjected to different tensile strengths; particularly, the effects of the strain rate on the tensile failure behavior of the materials can be seen. The PLA-Cu curves were consistent for all rates of applied tension, with evident yield points. The yield strength was equal to the maximum tensile strength, ranging from 45.23 to 60.39 MPa, which is proportional to the value of the stretching rate.

As shown in Figure 4a, after the tensile stress reached its peak, an instantaneous sharp decrease was not observed. When the tensile stress peak was reached, the specimen did not break and fail, but showed a period of gradual deterioration. More specifically, the load-bearing capacity slightly decreased when the maximum stress value was reached, then it rapidly decreased, exhibiting a clear stress decrease phenomenon, indicating that state of the PLA-Cu specimen was quasi-brittle.

The middle of the tensile stress–strain curve was concave. Conversely, the curve was slightly convex near the point of fracture, indicating that, during the period in which the material was being stretched, the microcracks and holes within the material underwent a process of opening–compacting–re-expansion. This is consistent with the conclusion of Yang Ge [23].

In the early stage of tensile loading, owing to the relatively small load and slow loading rate, a large number of axial microcracks, holes, and other defects were strongly affected by the tension, forming the first opening. When the strain reached 3%, the first opening was completed, a large number of existing defects were no longer open, and the specimen had entered the strengthening stage; this was associated with a concave opening on the stress–strain curve. When the strain reached 6.5%, because the load was close to the limit of the specimen, the specimen was about to yield, so new cracks occured until fracture, therefore entering the second phase, open. Next, a large number of cracks of macroscopic defects and reactions on the stress–strain curve for slightly upward convex phase, until eventually the curve, after extreme stress values, falls down and the specimen fractured.

Figure 4b shows the quasi-static compression stress–strain curve for the PLA-Cu material, which is similar to the quasi-static compression curve for the PLA material; additionally, both of them were found to be characteristic of hard and tough polymer materials. However, the difference between the two curves is that the curve for the PLA-Cu material revealed a compressive strength that was, on average, 38% higher than that of the PLA material.

It can be seen from Table 5 and Table 7 that the cross-section of PLA-Cu is consistently 10% larger than that of PLA, which was caused by the irregular surface of the printed sample caused by the filling of copper powder. However, the true cross-sectional area of the sample was used when calculating the stress and strain of the sample, so no error was caused to the stress–strain curve of the sample.

In summary, the yield stress and elastic modulus of the PLA and PLA-Cu materials increased with increasing strain rate. Under quasi-static tension, the PLA material had a critical strain rate that corresponded to a change from ductile to brittle. This is because in the PLA sample, with the increase of strain rate, the crack growth rate from the stress concentration point increased, so that the material without the stress concentration point has no time to stretch, and the sample was already broken.

### 3.2. Dynamic Mechanical Performance Test Results

Before the experiment, the diameter and height of the specimen (r×h) were measured. Table 9 lists the experimental parameters. The velocity of the impact bar was determined by calculating the time required for the rod to pass through the parallel light source.

The experimental results indicated that, under the condition of a strain rate of 10,200 s^−1^, the PLA material would fail once the impact bar reached 40.8 m/s. In the case of the PLA-Cu material, under the condition of a strain rate of 4150 s^−1^, crushing-induced failure was observed once the impact bar velocity reached 16.6 m/s.

**Figure 5:**
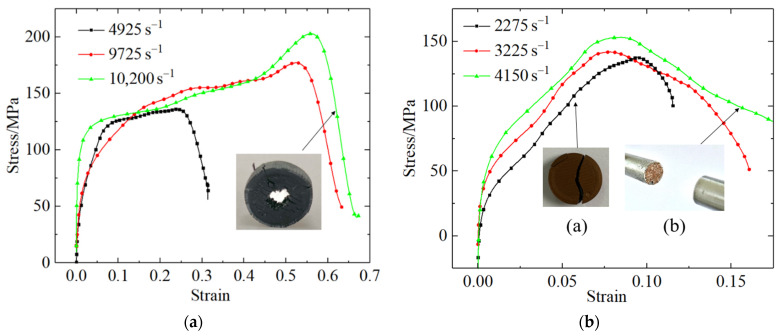
Dynamic stress–strain curves of (**a**) PLA; (**b**) PLA-Cu.

Each dynamic experiment was repeated three times, which was similar to the quasi-static condition. Figure 5 illustrates the average stress–strain curves for the PLA and PLA-Cu materials under the conditions of dynamic compression at different strain rates. Table 10 shows the error range of compressive strength.

**Figure 6:**
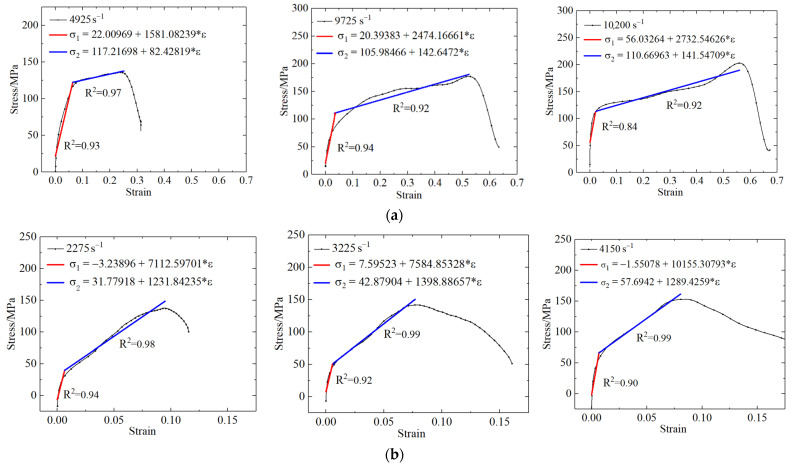
Schematic diagram of linear fitting of stress–strain curve of (**a**) PLA; (**b**) PLA-Cu.

Figure 5 depicts that the stress–strain curves for the PLA and PLA-Cu materials were nonlinear under the conditions of high strain rate loading, the process of which can be divided into three stages:

1. Linear elastic compression stage: In the elastic stage, the sample is impacted, and the internal void is compacted. The onset of strain causes the stress to rapidly increase, and the stress–strain curve exhibits a linear relationship.
2. Stress-hardening stage: the material enters the stress-hardening stage after yielding. The yield strength, elastic modulus, and hardening modulus of the material can be obtained by fitting the results associated with the linear elastic compression stage to those associated with the stress-hardening stage [17], as shown in Figure 6.
3. Failure and instability stage: as shown in Figure 5a, when the stress reaches the peak, the stress rapidly decreases as the strain increases, producing the failure stage, as shown in Figure 5b; when the peak stress is reached, the stress maintains a certain carrying capacity under the condition of increasing strain instead of rapidly decreasing. This stage is the slow strain-softening stage.

The similarities of the dynamic stress–strain curves for the two types of materials are as follows: With the increase of strain rate, the proportion of linear elastic compression stage became smaller and smaller because in the case of low strain rate, the impact rod brings less energy to the specimen, and the original space and cracks in the specimen are slowly closed, resulting in a longer compaction period. As the strain rate increased, the energy transferred to the specimen by the impact bar increased, and the closing speed of the initial crack inside the specimen increased. Thus, the linear stage decreased. This directly led to an increase in the slope of the stress–strain curve associated with the linear stage and an increase in the dynamic elastic modulus of the material.

Table 10 lists the mechanical parameters associated with dynamic compression of each material. The compressive strengths of the two materials were associated with the strain rate effect, as the peak stress under the condition of a high strain rate was much higher than that under the condition of a low strain rate. Furthermore, the results corresponding to the strain rate effect associated with dynamic compression loading and those obtained via the quasi-static compression experiment were consistent, as the compressive strength increased with increasing strain rate. However, the dynamic compressive strength of PLA was higher than that of PLA-Cu.

**Figure 7:**
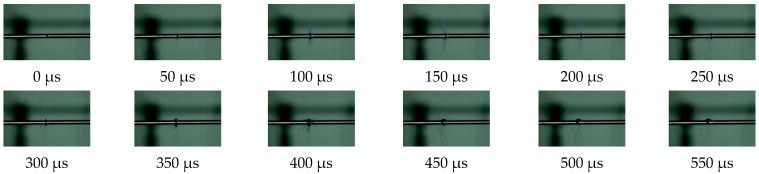
The middle part of PLA is broken (Strain rate is 10,200 s^−1^).

**Figure 8:**
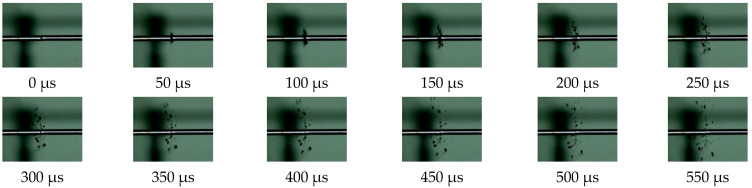
PLA-Cu crushing (Strain rate is 4150 s^−1^).

During the dynamic loading process, a high-speed camera was used to record changes in the specimen. A photograph was captured by the high-speed camera in intervals of 50 μs; each photograph was numbered and revealed the state of the specimen during the loading process. Figure 7 and Figure 8 show the deformation of the PLA and PLA-Cu samples that were subjected to dynamic loading. Considering the results presented in Figure 7 and Figure 8, in addition to the corresponding stress–strain curves, we can assume that the stress–strain relationship of each specimen was related to its failure during the loading process.

Figure 7 and Figure 8 show the dynamic loading failure processes for the two materials. Under the action of impact loading, the PLA sample did not experience instantaneous damage, as the failure was a dynamic process. Figure 7 shows that the PLA sample was compressed and deformed at 50 μs and exhibited cracks along the edges. Additionally, only a small portion of the material was separated from the sample body and scattered. The inset of Figure 5a demonstrates that at 400 μs the PLA sample was broken down by the incident bar in the center. Damage analysis of the sample revealed that the extent of damage increased as the impact speed increased; the damage was observed as a hole formed in the center of the sample.

In the case of the PLA-Cu sample, the impact began at 50 μs, and there was instantaneous failure of the specimen. Damage analysis revealed that an increase in stress was always accompanied by an explosive crushing process. The crushing process of the specimen was very short, i.e., approximately 150 μs, at which time the specimen was completely fractured.

The inset of Figure 5b illustrates the failure results for the PLA-Cu material according to strain rate. At low strain rates, only longitudinal crack failure modes appeares in the sample. Analysis of the recovered broken samples revealed that at high strain rates, under the action of compression loading, longitudinal cracks along the loading direction resulted in the formation of a large number of small fragments. This process was accompanied by the instantaneous release of stored energy in the specimen prior to failure.

### 3.3. Microscopic Morphology Analysis

To further analyze the microscale differences between the PLA and PLA-Cu samples, their microstructures were observed by SEM. Before observing the microstructure of the sample, we processed the sample in accordance with the JJG550-88 standard.

**Figure 9:**
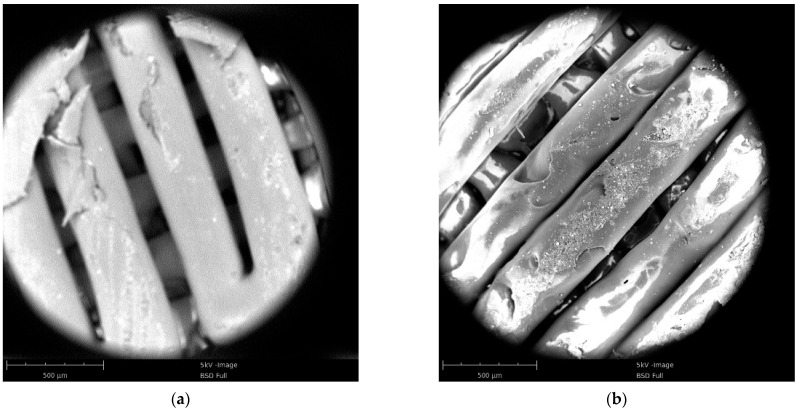

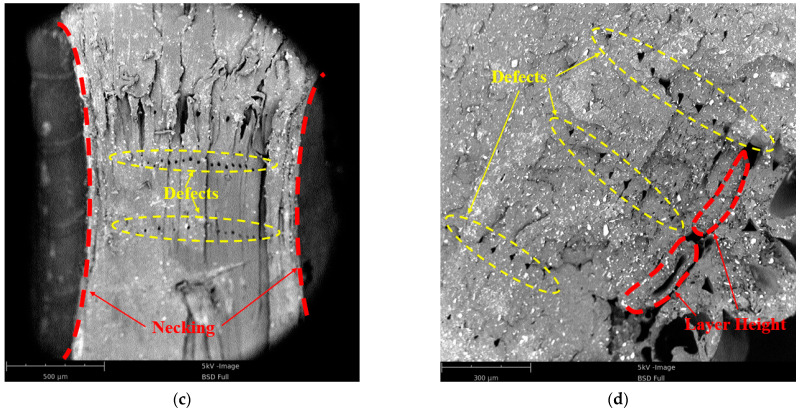
SEM images of 3D printed sample. (**a**) PLA sample surface; (**b**) PLA-Cu sample surface; (**c**) cross section of PLA sample; (**d**) cross section of PLA-Cu sample.

Figure 9 shows the resulting images of the 3D-printed sample. Because the printing parameters and conditions were the same, the printed samples of the two materials had the same structure, i.e., they were stacked layer by layer to form a grid crossing. Although the printing filling density was set to 100%, a gap could be observed between the printed filaments; this was an inherent feature of the 3D-printed samples.

Figure 9c,d are the cross sections of PLA and PLA-Cu tensile specimens, respectively. The comparative analysis shows that the biggest difference between the two is that the PLA tensile specimen had a necking phenomenon, while the PLA-Cu tensile specimen did not. At the same time, it can be seen that there are some defects in the two tensile samples. These defects were arranged in neat rows along the tensile loading direction, which had a slight impact on the tensile results.

However, there was a difference between the two materials: the internal voids of the 3D-printed PLA samples were larger than those of the PLA-Cu samples, and the PLA printed filaments had a uniform diameter and regular arrangement. The diameters of the filaments of the 3D-printed PLA-Cu samples were not constant, and irregularities were observed on the surface, indicating that the copper powder induced some defects in the 3D-printed sample. Additionally, the existence of these defects led to the emergence of concentrated stress points. In the case of the quasi-static tensile experiments, the copper powder filling negatively impacted the toughness of the PLA material, thereby accelerating the tensile fracture process of the PLA material.

**Figure 10:**
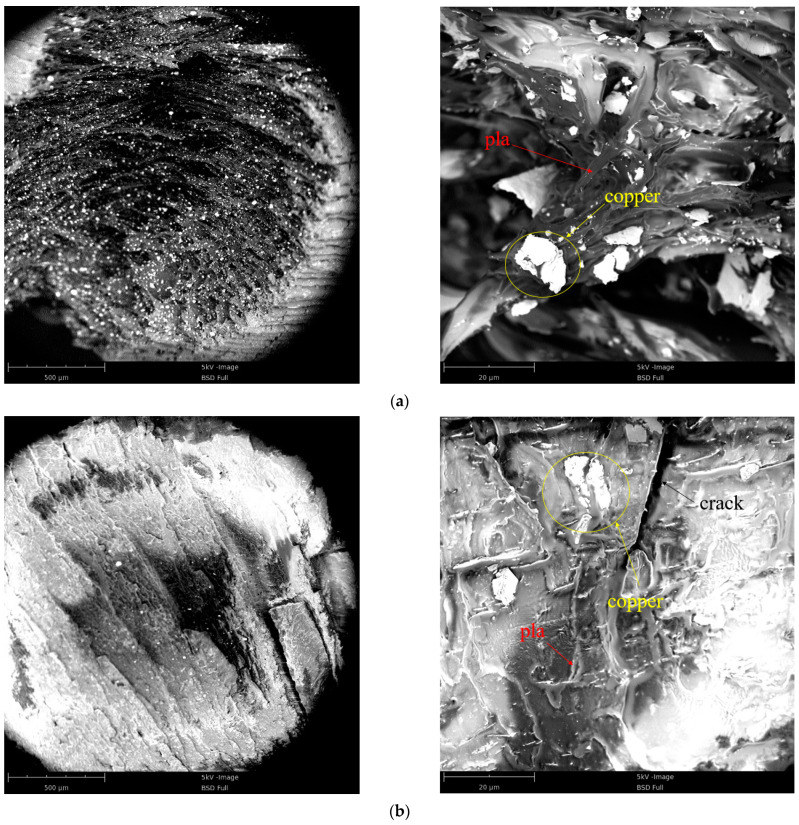
SEM image of sample. (**a**) Quasi-static compression crevasse; (**b**) dynamic impact cross section.

Figure 10 shows SEM images of the quasi-static compression specimen fracture and dynamic impact section of a PLA-Cu specimen. Figure 10a presents a fracture SEM image of the quasi-static compression sample; the white copper powder particles appeared as irregular lump-like fillers that were uniformly distributed throughout the PLA matrix. Because the failure mode of the material under uniaxial compression is similar to the failure mode of layer tearing, the PLA matrix exhibited appropriate viscosity.

Figure 10b demonstrates the cross section formed from the PLA-Cu specimen that was subjected to dynamic loading. The failure mode under this condition was significantly different from that under the condition of quasi-static compression. Particularly, the PLA-Cu material experienced a fully brittle fracture mode, wherein a large amount of plastic deformation occurred on the surface of the fracture. Additionally, a lamellar shedding phenomenon occurred. Considering these results and those obtained via quasi-static compression, we can assume that the presence of copper powder improves the static compressive strength of PLA material.

## 4. Constitutive Model

### 4.1. Parameter Fitting of ZWT Constitutive Model

**Figure 11:**
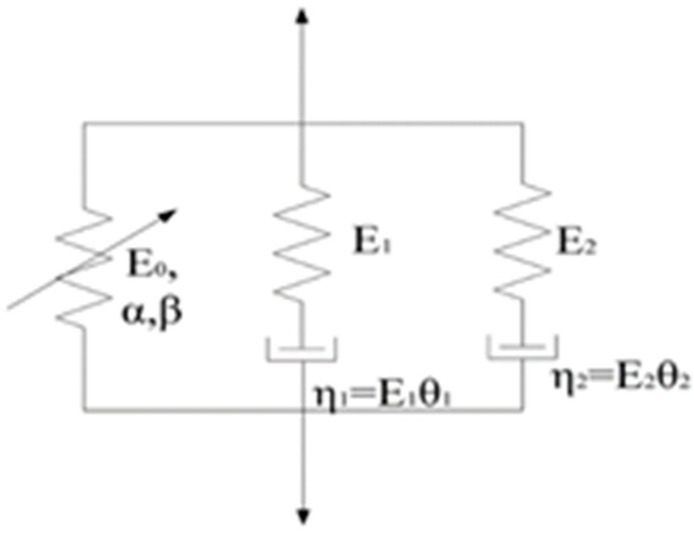
Schematic diagram of ZWT model [24].

The dynamic mechanical behavior of polymers can be described by applying the ZWT constitutive model [24,25,26,27,28]. The ZWT model is a nonlinear viscoelastic constitutive equation that can describe the mechanical properties of polymers within the range of viscoelastic deformation. This model can describe the mechanical behavior of a strain rate range of 10^−4^–10^3^ s^−1^ with appropriate accuracy. The ZWT model consists of a nonlinear spring and two Maxwell units, along with seven parameters. A schematic of the model is shown in Figure 11.

The ZWT model is expressed as follows:

(1) $$\sigma =E_{0}\varepsilon +\alpha \varepsilon^{2}+\beta \varepsilon^{3}+E_{1}\int_{0}^{t}\dot{\varepsilon }exp\left(\frac{-t-\tau }{\theta_{1}}\right)d\tau +E_{2}\int_{0}^{t}\dot{\varepsilon }exp\left(\frac{-t-\tau }{\theta_{2}}\right)d\tau$$

where *E*_0_, *α*, and *β* are nonlinear elastic constants; *E*_1_ and *E*_2_ are the linear elastic moduli at low and high strain rates, respectively; and *θ*_1_ and *θ*_2_ are the relaxation times at low and high strain rates, respectively.

The first three items in Equation (1) correspond to nonlinear springs, and are used to describe the nonlinear elastic response of the material. The fourth and fifth items correspond to two Maxwell elements and are used to describe the viscoelastic response of the material. The first integral term describes the viscoelastic response of the material under low strain rate loading, and the second integral describes the viscoelastic response of the material under high strain rate loading. Under constant strain rate loading, the integration of Equation (1) yields.

(2) $$\sigma =E_{0}\varepsilon +\alpha \varepsilon^{2}+\beta \varepsilon^{3}+E_{1}\theta_{1}\dot{\varepsilon }\left[1-exp\left(\frac{-t}{\theta_{1}}\right)\right]+E_{2}\theta_{2}\dot{\varepsilon }\left[1-exp\left(\frac{-t}{\theta_{2}}\right)\right]\;=E_{0}\varepsilon +\alpha \varepsilon^{2}+\beta \varepsilon^{3}+E_{1}\theta_{1}\dot{\varepsilon }\left[1-exp\left(\frac{-\varepsilon }{\dot{\varepsilon }\theta_{1}}\right)\right]+E_{2}\theta_{2}\dot{\varepsilon }\left[1-exp\left(\frac{-\varepsilon }{\dot{\varepsilon }\theta_{2}}\right)\right]$$

Under the condition of low strain rate loading, the high strain rate response term tends toward zero upon loading. Thus, the high strain rate integral term can be ignored, and the following equation can be obtained:

(3) $$\sigma =E_{0}\varepsilon +\alpha \varepsilon^{2}+\beta \varepsilon^{3}+E_{1}\theta_{1}\dot{\varepsilon }\left[1-exp\left(\frac{-\varepsilon }{\dot{\varepsilon }\theta_{1}}\right)\right]$$

Under the condition of high strain rate loading, the incident bar rapidly impacts the specimen, and the low-frequency response is too slow to result in relaxation. Therefore, the first Maxwell element can be treated as a spring, and the elastic modulus is *E*_1_. The following equation can be obtained:

(4) $$\sigma =(E_{0}+E_{1})\varepsilon +\alpha \varepsilon^{2}+\beta \varepsilon^{3}+E_{2}\theta_{2}\dot{\varepsilon }\left[1-exp\left(\frac{-\varepsilon }{\dot{\varepsilon }\theta_{1}}\right)\right]$$

Note that the influence of room temperature changes on the experimental results was not considered when fitting the parameters of the ZWT constitutive equation, because all of the experiments in this study were performed concurrently in the same place.

In the cases of both materials, i.e., the PLA and PLA-Cu materials, two quasi-static stress–strain curves with strain rates of 0.083 s^−1^ and 0.033 s^−1^, respectively, were selected and subtracted to obtain the corresponding stress–strain curves containing only *E*_1_ and *θ*_1_. The respective relationships between stress change and strain were obtained by fitting the stress change curve according to Equation (2). Then, *E*_1_ and *θ*_1_ were substituted into Equation (3), and *E*_0_, *α*, and *β* were obtained by fitting each stress–strain curve with a strain rate of 0.083 s^−1^. Lastly, the above-mentioned five parameters were substituted into Equation (4) to obtain *E*_2_ and *θ*_2_ by applying stress–strain curve fitting to the high strain rate results. The high strain rates for the PLA and PLA-Cu materials were 4925 s^−1^ and 2275 s^−1^, respectively.

**Figure 12:**
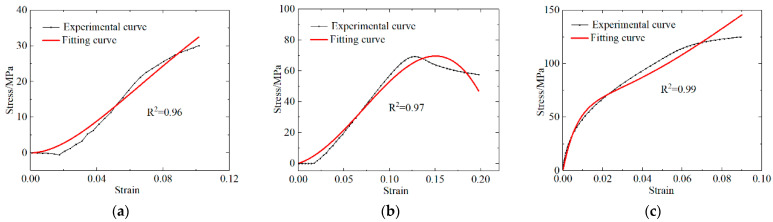
Comparison of PLA experimental curve and fitting curve. (**a**) Low strain rate stress difference; (**b**) low strain rate; (**c**) high strain rate.

**Figure 13:**
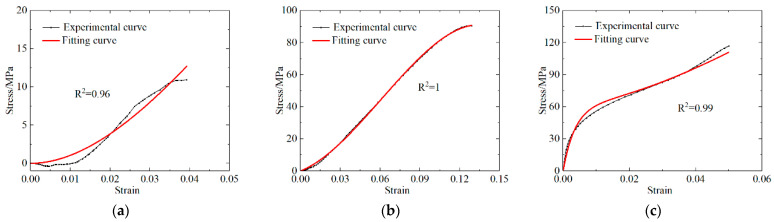
Comparison of PLA-Cu experimental curve and fitting curve. (**a**) Low strain rate stress difference; (**b**) low strain rate; (**c**) high strain rate.

The experimental and simulated curves were comparatively analyzed for both materials, and the results are shown in Figure 12 and Figure 13. The ZWT constitutive parameters for the PLA and PLA-Cu samples were also obtained, and the results are presented in Table 11.

The numerically simulated results demonstrate that the ZWT constitutive equation can describe the mechanical behavior of PLA materials with a strain in the range of 0–14% under quasi-static conditions, and in the range of 0–7% under dynamic conditions. In the case of the PLA-Cu material, the ZWT constitutive equation can describe the mechanical behavior of the strain in the range of 0–13% under quasi-static conditions, and in the range of 0–5% under dynamic conditions.

In Figure 10 and Figure 11, R^2^ is the correlation coefficient, and the fitted R^2^ was calculated to be above 95%, which is approximately 1, indicating that the ZWT constitutive equation can describe the elasticity and yield stage of the two materials at the corresponding strain rate with reasonable accuracy. The results revealed that the materials exhibit the characteristics of a viscoelastic material and that the established ZWT viscoelastic constitutive model can accurately predict the experimental results.

### 4.2. Constitutive Model Verification

The parameters of the ZWT constitutive model (Table 11) were obtained by fitting two stress–strain curves under quasi-static conditions and simulating one stress–strain curve that was generated under the condition of a high strain rate. To verify the feasibility of the parameters, the theoretically predicted and experimental values of the other two groups of high-strainrate stress–strain curves were compared.

**Figure 14:**
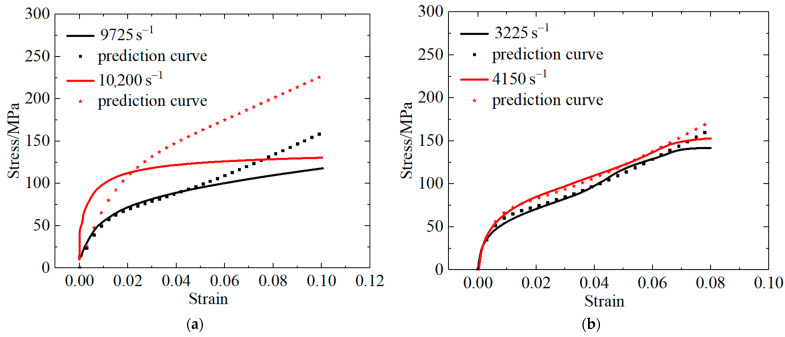
Comparison of theoretical prediction curve and experimental curve of (**a**) PLA; (**b**) PLA-Cu.

The parameters of the ZWT constitutive model (Table 11) were substituted into Equation (4) to obtain the prediction curve equation for high strain rates. The results of the predicted and experimental curves were subsequently compared, as shown in Figure 14.

Comparative analysis of the predicted and experimental curves revealed that, in the case of the PLA material, under the condition of a strain rate of 9725 s^−1^ and strain below 5%, the predicted curve agrees well with the experimental curve, indicating that the predicted curve can accurately describe the mechanical behavior of materials with a strain of less than 5%.

When the strain rate was 10,200 s^−1^, the predicted curve significantly differed from the experimental curve; this is because the linear stage of the stress–strain curve became nearly perpendicular to the strain-hardening stage as the strain rate increased. Consequently, the curve equation did not fit the exponential equation. Thus, when the strain rate exceeds 10,200 s^−1^, the constitutive equation is not applicable.

Figure 14b depicts that the predicted values of the two stress–strain curves for the PLA-Cu material did not significantly differ from the experimental values and that the predicted curves can describe the dynamic mechanical behavior of the material under the condition of strain within 7%.

## 5. Conclusions

The static and dynamic mechanical properties of PLA and PLA-Cu material were tested using a universal material testing machine and an SHPB experimental device. The following conclusions were drawn.

1. Under quasi-static conditions, the PLA material is strongly influenced by the strain rate. Specifically, when the strain rate was increased, the strength of the material increased, and the toughness and ductility decreased. The entire specimen also exhibited a change from tough to brittle beginning at the critical strain rate value of 0.033 s^−1^. SEM revealed that the presence of copper powder induced defects on the surface of the 3D-printed PLA samples. These defects negatively impacted the toughness of the material, accelerating the onset of tensile fracture and improving the compressive strength of the PLA material.

2. The stress–strain curves that were obtained for the PLA and PLA-Cu materials under dynamic loading conditions revealed the existence of a linear elastic compression stage, a stress-hardening stage, and a failure and instability stage; the curves also confirmed nonlinearity. Numerical simulation of the linear elastic stage and stress-hardening stage revealed that the copper powder substantially increased the dynamic linear elastic modulus and hardening modulus of the PLA material. Additionally, the photographs obtained via high-speed camera revealed that the presence of copper powder caused the PLA material to become non-ductile. Furthermore, post-impact specimen failure always occurred as an explosive crushing process.

3. The dynamic mechanical behavior of the PLA material confirmed its viscoelastic property. The ZWT constitutive model was used to fit the experimental results; seven parameters of the constitutive models of the PLA and PLA-Cu specimen were obtained, and the parameters were verified. We found that the model can describe the mechanical properties of the PLA-Cu material under the condition of a strain of less than 7%. However, this constitutive equation is not applicable when the strain rate exceeds 10,200 s^−1^.

## Figures and Tables

**Table 1:** Physical properties of PLA.

Attributes	Testing Method	Units	Test Conditions	Typical Value	Attributes
Density	ASTM D792	g/cm^3^	-	1.24	Density
The melt flow rate	ASTM D1238	g/10 mn	210 °C, 2.16 kg	7	The melt flow rate
Melt density	-	g/cm^3^	230 °C	1.08	Melt density
Melting point	-	°C	-	155–170	Melting point

**Table 2:** Mechanical properties of PLA.

Attributes	Testing Method	Units	Test Conditions	Typical Value
Tensile strength	ASTM D882	MPa	Break	53
			yield	60
Tensile modulus		GPa	-	3.5
Cantilever notch test	ASTM D256	J/m	-	16

**Table 3:** Experimental equipment.

Name	Model	Source	City	Country
3D Printer	Ultimaker S5 3D	Ultimaker Holding B.V.	Utrecht	The Netherlands
Scanning Electron Microscope (SEM)	PhenomTM	Phenom-World BV	Eindhoven	The Netherlands
SHPB	φ12	Harbin Institute of Technology	Harbin	China
High-Speed Camera	FASTCAM SA-X2	Photron	Tokyo	Japan
Universal Material Testing Machine	WDW-20	Shandong Zhongyi Instruments Co.	Shandong	China

**Table 4:** Fixed parameters of 3D printing.

Parameter	Nozzle Diameter	Printing Speed	Printing Layer Height	Printing Method
Numerical Value	0.4 mm	20 mm/min	0.06 mm	Grid Cross

**Table 5:** PLA quasi-static tensile test data.

Tensile Speed (mm/min)	Section Size (b × d/mm)	Elongation at Break	Yield Stress/MPa	Tensile Strength/MPa	Modulus of Elasticity/MPa
2	5.08 × 2.02	145%	55.95 (*±1.12*)	144.68 (*±5.10*)	534.56 (*±10.70*)
5	5.06 × 2.02	162%	56.38 (*±2.56*)	186.99 (*±16.64*)	588.17 (*±26.70*)
10	5.06 × 2.04	145%	63.84 (*±0.48*)	141.43 (*±15.20*)	586.55 (*±4.41*)
20	5.10 × 2.06	19.5%	65.01 (*±1.47*)	65.01 (*±3.15*)	596.08 (*±13.47*)
50	5.04 × 2.10	23.9%	69.95 (*±0.65*)	69.95 (*±1.77*)	617.78 (*±5.74*)

**Table 6:** PLA quasi-static compression experimental data.

Compression Speed (mm/min)	Strain Rate (s^−1^)	Section Size (r × h/mm)	Compression Strength/MPa	Modulus of Elasticity/MPa
2	0.003	4.97 × 10.38	50.01 (*±5.30*)	333.53 (*±35.34*)
5	0.008	4.95 × 10.30	42.78 (*±3.02*)	242.22 (*±17.10*)
10	0.016	4.99 × 10.36	44.32 (*±2.65*)	251.10 (*±15.01*)
20	0.033	4.96 × 10.30	54.76 (*±0.16*)	380.61 (*±1.00*)
50	0.083	4.99 × 10.30	69.34 (*±2.56*)	672.41 (*±24.82*)

**Table 7:** Quasi-static tensile test data of PLA-Cu.

Tensile Speed (mm/min)	Section Size (b × d/mm)	Elongation at Break	Yield Stress/MPa	Modulus of Elasticity/MPa
2	5.30 × 2.22	7.65%	45.23 (*±1.31*)	585.82 (*±16.96*)
5	5.30 × 2.18	8.58%	52.63 (*±2.93*)	616.56 (*±34.32*)
10	5.26 × 2.22	8.35%	53.22 (*±0.32*)	655.12 (*±3.94*)
20	5.26 × 2.26	9.22%	60.37 (*±1.99*)	670.67 (*±22.11*)
50	5.28 × 2.24	9.53%	60.39 (*±2.49*)	640.08 (*±26.39*)

**Table 8:** Quasi-static compression experimental data of PLA-Cu.

Compression Speed (mm/min)	Strain Rate (s^−1^)	Section Size (r × h/mm)	Compression Strength/MPa	Modulus of Elasticity/MPa
2	0.003	5.10 × 10.18	74.94 (*±2.79*)	764.35 (*±28.46*)
5	0.008	5.13 × 10.20	81.21 (*±0.71*)	653.35 (*±5.71*)
10	0.016	5.13 × 10.22	87.80 (*±0.88*)	672.42 (*±5.90*)
20	0.033	5.13 × 10.22	87.59 (*±1.98*)	686.67 (*±15.52*)
50	0.083	5.12 × 10.28	90.61 (*±5.51*)	760.52 (*±46.24*)

**Table 9:** SHPB experimental scheme.

Materials	Strain Rate (s^−1^)	Initial Size (r × h/mm)	Air Chamber Pressure (MPa)	Movement Time of Impact Bar (μs)	Impact Bar Velocity (m/s)	State of the Specimens
PLA	4925	12.04 × 4.28	0.5	2030.2	19.7	Cracking
	9725	11.98 × 4.28	1.2	1029.5	38.9	breakdown
	10,200	12.02 × 4.28	1.4	981.1	40.8	broken
PLA-Cu	2275	12.22 × 4.20	0.05	4382.1	9.1	Cracking
	3225	12.16 × 4.20	0.125	3109.5	12.9	Cracking
	4150	12.18 × 4.20	0.2	2409.3	16.6	Crushing

**Table 10:** SHPB experimental scheme.

Materials	Strain Rate (s^−1^)	Yield Strength (MPa)	Yield Strain	Elasticity Modulus	Compression Strength (MPa)	Hardening Modulus
PLA	4925	122.48	6.4%	1581.08	135.64 (*±12.21*)	82.43
	9725	111.17	3.6%	2474.16	177.11 (*±25.41*)	142.65
	10,200	114.27	2.3%	2732.55	202.99 (*±25.92*)	114.55
PLA-Cu	2275	40.53	0.71%	7112.6	137.59 (*±4.14*)	1231.84
	3225	49.82	0.71%	7584.85	141.75 (*±8.04*)	1398.89
	4150	68.48	0.83%	10,155.31	152.93 (*±6.47*)	1286.42

**Table 11:** Parameters of ZWT constitutive equation.

Materials	E_1_/MPa	θ_1_/s	E_2_/MPa	θ_2_/μs	E_0_/MPa	α/MPa	β/MPa
PLA	1207.9	1.3082	9450.3	1.369 × 10^−6^	−1014.5	11,699	−41,340
PLA-Cu	2077.7	1.6661	16,721	1.313 × 10^−6^	−1746.9	17,273	−67,812

## Data Availability

The data presented in this study are available on request from the corresponding author.
